# Cognitive screening and rehabilitation after cardiac arrest: only a few hurdles to take

**DOI:** 10.1007/s12471-023-01838-4

**Published:** 2023-12-12

**Authors:** Janine A. van Til, Martin E. W. Hemels, Jeannette Hofmeijer

**Affiliations:** 1 Department of Health Technology and Services Research, Technical Medical Centre, University of Twente, Enschede, The Netherlands; 2 Department of Cardiology, Rijnstate Hospital, Arnhem, The Netherlands; 3 Department of Cardiology, Radboud University Medical Centre, Nijmegen, The Netherlands; 4 Department of Neurology, Rijnstate Hospital, Arnhem, The Netherlands; 5 Department of Clinical Neurophysiology, Technical Medical Centre, University of Twente, Enschede, The Netherlands

**Keywords:** Cardiac arrest, Cognitive impairment, Screening, Guidelines, Implementation

## Abstract

Dutch and European guidelines recommend systematic screening for cognitive and emotional impairments in cardiac arrest survivors. We aimed to clarify opinions on cognitive screening and rehabilitation, identify barriers and facilitators for implementation in the Netherlands, and arrive at recommendations in this field. We conducted 22 semi-structured interviews with various stakeholders using the Tailored Implementation in Chronic Diseases checklist. There is broad-based acknowledgement of the relevance of cognitive impairment and a positive attitude regarding early cognitive screening among health professionals and patients. Barriers to implementation include a lack of practical recommendations on how, where and when to screen, insufficient knowledge of cognitive consequences of cardiac arrest, insufficient collaboration and knowledge sharing among different specialties within hospitals, insufficient resources, and insufficient evidence of the effectiveness of screening and therapy to justify financial compensation. Most of the identified barriers to implementation are solvable: national guidelines need practical recommendations and knowledge gaps among healthcare workers can be bridged by in-hospital collaboration. Fulfilling these requirements should be sufficient for the implementation of simple screening and tailored advice. More extensive cognitive rehabilitation therapy needs stronger evidence of efficacy in order to warrant stronger guideline recommendations and financial reimbursement.

## Introduction

The annual incidence of out-of-hospital cardiac arrest (OHCA) in Europe ranges from 67 to 170 cases per 100,000 inhabitants per year [1]. Survival after OHCA has improved substantially over the past few decades [2]. In the Netherlands alone, 3395 individuals with OHCA were hospitalised in 2021, of whom 60% were discharged from hospital [3]. In sharp contrast to the increased survival, the neurological outcome of survivors has changed only marginally. Long-term disturbances of cognition and mood have been recognised in 50–100% of all survivors [2, 4]. As a result, half of all patients cannot resume their daily activities and three-quarters demonstrate impaired social participation [5]. Cognitive impairments are strongly related to reduced quality of life [6].

For these reasons, Dutch and European guidelines recommend systematic screening for cognitive and emotional impairments in all cardiac arrest survivors [7, 8]. Nevertheless, cognitive functioning, mood and anxiety are addressed infrequently and not systematically. We aimed to clarify opinions on cognitive screening and rehabilitation after cardiac arrest, identify barriers and facilitators for implementation in the Netherlands, and to arrive at recommendations for implementation or new studies in this field.

## Methods

We conducted 22 semi-structured interviews with 11 healthcare professionals (cardiologists, rehabilitation physicians, specialised nurses and an occupational therapist), six patients (including some with and some without cognitive impairment), two managers, three policy makers and one health insurer. We used the Tailored Implementation in Chronic Diseases checklist [9] to develop the interview guide and to structure deductive coding of interview transcripts.

This study was performed in line with the principles of the Declaration of Helsinki. Approval was granted by the Ethics Committee of the University of Twente.

## Results

All interviewees acknowledged the relevance of long-term cognitive impairment and the importance of including some form of cognitive screening in cardiac rehabilitation programmes. The identified barriers to and facilitators for implementation are summarised in Table 1. The main identified facilitators are the perceived relevance of addressing cognitive impairments after cardiac arrest by all stakeholders and a strong belief, particularly among healthcare professionals, that systematic cognitive screening and rehabilitation are compatible with current care. Another major facilitator is the availability of local protocols from hospitals and rehabilitation centres that have already included systematic cognitive screening in cardiac rehabilitation programmes.

**Table 1:** Barriers and facilitators for implementation of cognitive screening and rehabilitation in cardiac rehabilitation programmes in the Netherlands

Barriers	Facilitators	Proposed next steps
*Guideline factors*		
– Lack of practical instructions in (inter)national guidelines on how, where and when to screen – Lack of evidence of efficacy	– Cognitive screening recommended by (inter)national guidelines – Local protocols available	– Studies into effects of cognitive screening and rehabilitation – Include practical instructions in guidelines
*Individual health professional factors*		
– Lack of knowledge of cognitive consequences of cardiac arrest – Lack of familiarity with cognitive screening among cardiologists	– Positive attitude towards cognitive screening – Necessary skills easy to train	– Develop a training programme for recognition of cognitive impairment, screening and referral
*Patient factors*		
– Lack of awareness of own cognitive impairment – Burden of early cognitive screening	– Positive attitude towards cognitive screening	– Implement screening in stable patients; avoid screening in the first few weeks
*Professional interactions*		
– Current referral processes not optimal – Brain specialist or specialised nurse not involved early – Uncertainty about responsibilities and roles – Poor structural collaboration between cardiology department and other disciplines	– Positive attitude towards collaboration between hospital departments – Existing knowledge in other hospital departments	– Implement multidisciplinary consultation – Tighten collaboration
*Incentives and resources*		
– (Trained) Personnel constraints – Time constraints – Financial constraints – Not included in ‘diagnosis-treatment combination’	– Inclusion in the guideline to facilitate reimbursement	– Include in ‘diagnosis-treatment combination’ – Talk with health insurers – Provide evidence of efficacy
*Capacity for organisational change*		
– High workload	– Willingness of managers	– Managerial and administrative support
*Social, political and legal factors*		
– Negotiating procedures with health insurers	– Legislation not an absolute barrier to implementation	

Note: These barriers and facilitators were identified during interviews with three cardiologists, four rehabilitation physicians, three specialised nurses, one occupational therapist, two managers, three policy makers, six patients and one health insurer

The main identified barriers include a lack of knowledge of the cognitive consequences of cardiac arrest among patients and healthcare professionals. Patients also expressed concerns about the possible impact of early screening, suggesting that the benefits should outweigh the additional burden its puts on patients and family in the first weeks following a cardiac arrest. Healthcare professionals pointed out the lack of practical instructions in guidelines on how, when and where to perform cognitive screening. Healthcare professionals at cardiology departments indicated not having the knowledge and skills to recognise and address cognitive impairment. This comes together with insufficient structural cooperation between the cardiac, neurology and (neuro)psychology departments within a hospital. Both healthcare professionals and managers mentioned the limited availability of resources, mainly time and trained personnel, as barriers to the implementation of systematic cognitive screening. Specifically, healthcare professionals mentioned the time required to identify patients eligible for cognitive screening, performing the screening itself, and the associated administrative tasks as examples of time-consuming activities that would be added to their current workload. Finally, the lack of strong evidence regarding the effectiveness of cognitive screening and rehabilitation therapy has been identified as a barrier to implementation, as well as to strengthening recommendations in guidelines. The health insurer indicated that evidence and strong recommendations would facilitate reimbursement.

## Discussion

The results of our interviews indicate that a broad range of stakeholders acknowledge the relevance of cognitive and emotional impairments after cardiac arrest and the importance of including some form of cognitive screening in cardiac rehabilitation programmes. All stakeholders had a positive attitude towards cognitive screening and rehabilitation, and healthcare professionals indicated that these are, in principle, compatible with current practices.

Current (inter)national guidelines on cardiac rehabilitation represent the main identified barrier to implementation. This is remarkable, since both the Dutch and European guidelines recommend cognitive screening and referral for cognitive rehabilitation therapy in the case of suspected cognitive impairment [7, 8]. However, healthcare professionals indicate the lack of practical instructions on various aspects of the recommendation, particularly regarding the mode of screening, the target patient group, the setting in which screening should take place, and timing. This gap is partly filled by the availability of local protocols from hospitals and rehabilitation centres that have already implemented systematic screening and cognitive rehabilitation, which could be transferred to other hospitals. Examples include the workbook *Rehabilitation after Resuscitation* and the compact intervention *Stand still …, and move on* [10, 11]. One of the recommendations based on the expertise of these centres is to implement systematic cognitive screening by means of the Montreal Cognitive Assessment (MoCA) test at 3 months after cardiac arrest, with referral to a rehabilitation specialist for tailored advice for patients with a MoCA score < 26.

Another barrier is the lack of knowledge of the cognitive consequences of cardiac arrest. In particular, healthcare professionals at cardiology departments regretted having insufficient knowledge and skills to recognise and address cognitive impairment. This is in line with a previous Dutch study, in which 31% of rehabilitation specialists mentioned a lack of expertise among cardiologists regarding cognitive impairment [12]. This barrier is enhanced by an apparent lack of structural cooperation between healthcare professionals from different disciplines treating patients after cardiac arrest. Both we and others found that improved collaboration between cardiology and neurology departments is perceived as vital for successful implementation of brain training in cardiac rehabilitation programmes [12].

Most patients expressed the significance of cognitive impairment in their daily lives and the challenging path they had to navigate to seek the necessary assistance. In certain instances, earlier screening and subsequent diagnosis could have averted some of these issues. However, the patients also indicated that both they and their families experienced high strain levels during the first weeks after cardiac arrest. Therefore, the benefits of adding cognitive screening during these weeks should be balanced against its burden on the patients. To ensure optimal implementation of screening from the patient’s perspective, it is essential to select a screening instrument that is relatively short and easy to administer, exercise caution in timing and provide adequate aftercare.

Other barriers to implementation are related to the availability of resources, especially time and trained personnel. Reimbursement by the health insurer would facilitate inclusion in the ‘diagnosis-treatment combination’ (care path) and provide financial support for implementation. Health insurers indicate that stronger recommendations in guidelines would facilitate reimbursement, whereas policy and guideline makers mention the lack of strong evidence of efficacy as barriers for recommendations in guidelines. This identical detrimental cycle is applicable to implementation of cognitive screening in related fields, such as in stroke care [13]. Stronger evidence would thus support all aspects of implementation.

## Conclusion

We conclude that systematic cognitive screening and rehabilitation after cardiac arrest are supported by a broad-based positive attitude among healthcare professionals and patients. Most identified barriers for implementation are solvable: national guidelines need practical recommendations and knowledge gaps among healthcare workers can be solved by in-hospital collaboration. These should be sufficient for implementation of simple screening and tailored advice. More extensive cognitive rehabilitation therapy needs stronger evidence of efficacy in order to warrant stronger recommendations and financial reimbursement.
